# A case report of IgG4-related disease: an insidious path to the diagnosis through kidney, heart and brain

**DOI:** 10.1186/s12882-019-1587-4

**Published:** 2019-11-21

**Authors:** Giorgia Comai, Vania Cuna, Benedetta Fabbrizio, Elena Sabattini, Ornella Leone, Francesco Tondolo, Andrea Angeletti, Maria Cappuccilli, Rocco Liguori, Gaetano La Manna

**Affiliations:** 1 Unit of Nephrology, Dialysis and Transplantation, Department of Experimental Diagnostic and Specialty Medicine, University of Bologna, S. Orsola Malpighi Hospital, Via G. Massarenti 9 (Pad. 15), 40138 Bologna, Italy; 2 Unit of Oncology and Transplant Pathology, Department of Experimental Diagnostic and Specialty Medicine, University of Bologna, S. Orsola Malpighi Hospital, Via G. Massarenti 9 (Pad. 18), 40138 Bologna, Italy; 3 Unit of Hemolymphopathology, Department of Hematology & Oncology, University of Bologna, S. Orsola Malpighi Hospital, Via G. Massarenti 9 (Pad. 8), 40138 Bologna, Italy; 4 IRCCS Institute of Neurological Sciences of Bologna and Department of Biomedical and Neuromotor Sciences, University of Bologna, Via Altura 3, 40139 Bologna, Italy

**Keywords:** B-lymphocytes, IgG4-related disease, Kidney, Nervous system, T-lymphocytes

## Abstract

**Background:**

IgG4-related disease, described around the years 2000 as a form of autoimmune pancreatitis, is now increasingly accepted as a systemic syndrome. The diagnosis is based on both comprehensive and organ-specific criteria. For the kidney, Mayo clinic classification and the guidelines of the Japanese Nephrology Society are used. Ultimately, together with parameters that characterize every organ or apparatus involved, the key element is the confirmation of growing levels of IgG4 in blood or in tissues.

**Case presentation:**

We describe a male patient with chronic renal failure associated to hypertension without proteinuria. IgG4-related disease was diagnosed through renal biopsy. After an initial positive response to steroids, he presented tinnitus, and histological assessment showed cerebral and subsequently cardiac damage, both IgG4-related. This case appears unique for the type of histologically documented cardiac and neurological parenchymal involvement, and at the same time, exemplifies the subtle and pernicious course of the disease. Frequently, blurred and non-specific signs prevail. Here, kidney damage was associated with minimal urinary findings, slowly progressive renal dysfunction and other factors that can be equivocated in the differential diagnosis. Neurological involvement was represented by tinnitus alone, while cardiac alterations were completely asymptomatic.

**Conclusions:**

This report is representative of the neurological and cardiac changes described in the literature for IgG4-related disease, which may be correlated or not with the renal form and highlights the need, in some cases, of targeted therapeutic approaches. In addition to glucocorticoids, as in this case, rituximab may be necessary.

## Background

IgG4-Related Disease (IgG4-RD) is a systemic immune-mediated fibroinflammatory condition. Several well-known illnesses (Mikulicz’s disease, Riedel’s thyroiditis, Morbus Ormond, Kuttner’s tumor), once considered unrelated, are now unified under this systemic disorder [1]. The epidemiology is not well defined: it usually affects adults from middle-age onwards, predominantly male.

The pathogenesis of IgG4-RD can be highly variable. The presentation of an unknown antigen by B-cells and plasmablasts to CD4+ T-lymphocytes triggers the local production of fibrogenic cytokines, namely IL-1β, IFN-γ and TGF- β, which contribute to spread the fibrosis to different organs. For unknown reasons, the previous interaction, involving CD4+ Tfh2 (IL-4 and IL-10), induces a switch to IgG4 production within lymph node germinal centers. Different autoantigens, like proteins expressed in bile duct epithelia and in pancreatic acinar cells, have been proposed as targets/triggers in IgG4-RD patients, although they are not found in all the organs potentially involved [2].

Both B and T-cells are central in IgG4-RD pathogenesis, as demonstrated by the efficacy of B-cell depletion therapy [3, 4].

The most common affection is lymphoplasmacytic sclerosing pancreatitis, but many others can be listed relating to this disease: dacryoadenitis and Mikulicz disease, lymph node adenopathies, aortitis, arteritis, sclerosing cholangitis, tubulointerstitial nephritis, membranous nephropathy and retroperitoneal fibrosis [1]. Both central and peripheral nervous system are implicated in IgG4-RD, with a large number of different signs and symptoms, depending on the nervous structure injured [5, 6].

Unfortunately, the heterogeneity and the vagueness of these symptoms do not permit a clear and standardized identification, causing many delays and difficulties in the diagnosis.

The current approach to the diagnosis of IgG4-RD is based on a combination of comprehensive and organ-specific diagnostic criteria. Concerning the kidney, the Mayo clinic classification and the guidelines of the Japanese Nephrology Society are applied [7–9].

We describe here a particular case report, somehow unique, characterized by both neurological and cardiological involvement, with slightly different features from the currently available literature [6].

## Case presentation

A 51-year-old man with a history of hypertension, nephrolithiasis and benign prostatic hyperplasia was admitted to our hospital because of progressive chronic kidney disease with worsening proteinuria and low serum complement levels. The patient’s anamnesis was negative for nephrotoxic drugs and included asthmatic bronchitis, treated with desensitization immunotherapy, inguinal and submandibular lymphadenopathy, with a histological pattern described as reactive follicular hyperplasia, and a recent suspected connective tissue disease. At the time of admission, the patient’s general conditions were good. Laboratory tests revealed the following: elevated serum creatinine levels (183 μmol/L), high serum total IgG and IgG4 levels (2882 mg/dL and 1853 mg/dL, respectively), high serum IgE levels (4442 UI/mL) without eosinophilia, low serum complement levels (72.8 mg/dL), elevated free kappa and lambda chains with normal ratio, negative serum and urine immunofixation test, and a proteinuria of 340 mg/24 h without hematuria. Blood pressure was under control and the general physical examination was unremarkable.

**Fig. 1:**
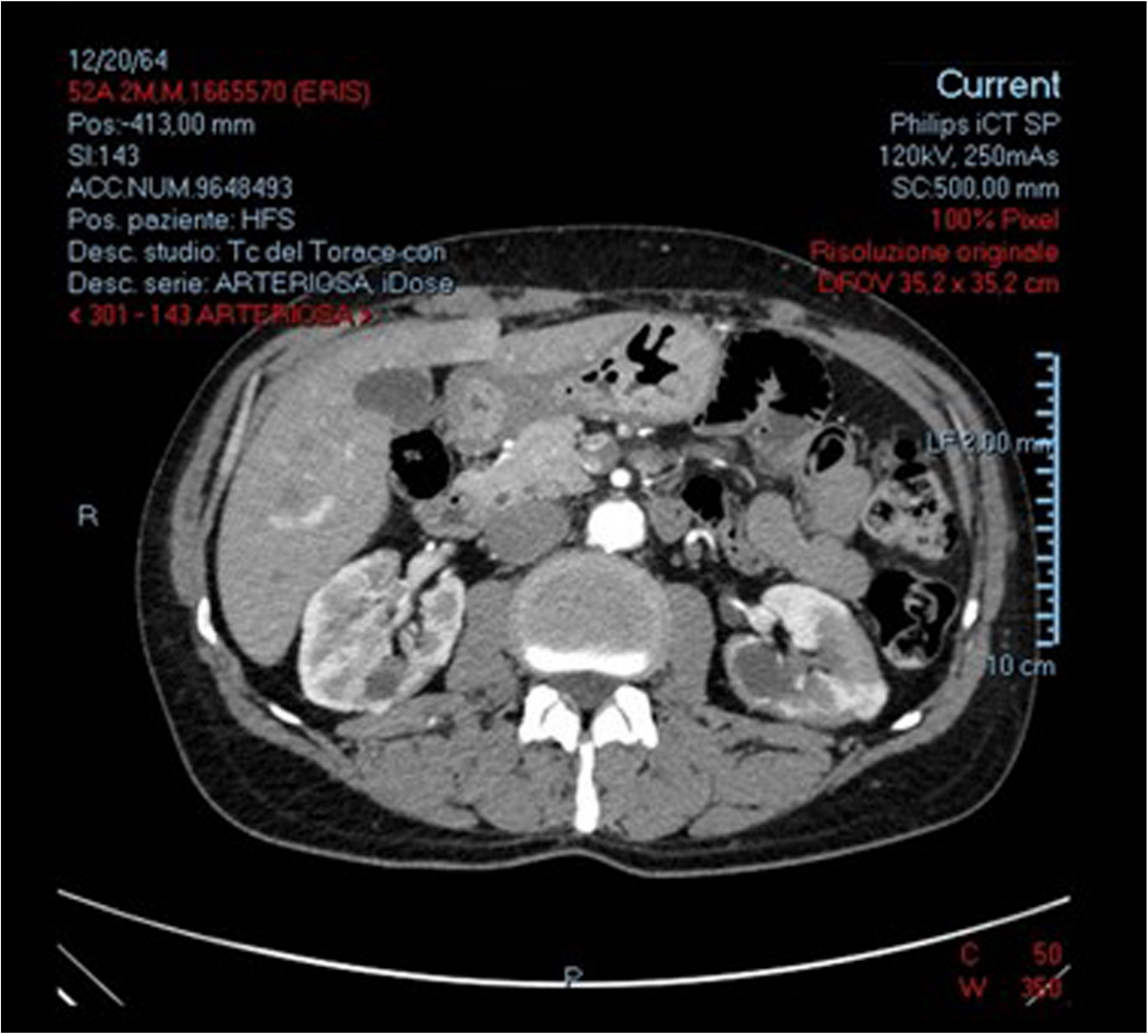
Bilateral renal low density lesions on enhanced computed tomography (CT)

Abdominal ultrasound displayed both enlarged kidneys (12.5 cm longitudinal diameter for the right kidney, 14 cm for the left) showing hyperechogenic cortex with soft margin and incisures resulting from past lesions, without any other abdominal abnormalities. Enhanced computed tomography (CT) highlighted multiple renal low-density lesions (Fig. 1).

Owing to the patient’s history of lymphadenopathy, PET/CT was performed. It showed tenuously submandibular, retroperitoneal and left inguinal hypermetabolic adenopathy, without a clear evidence of infectious focus.

**Fig. 2:**
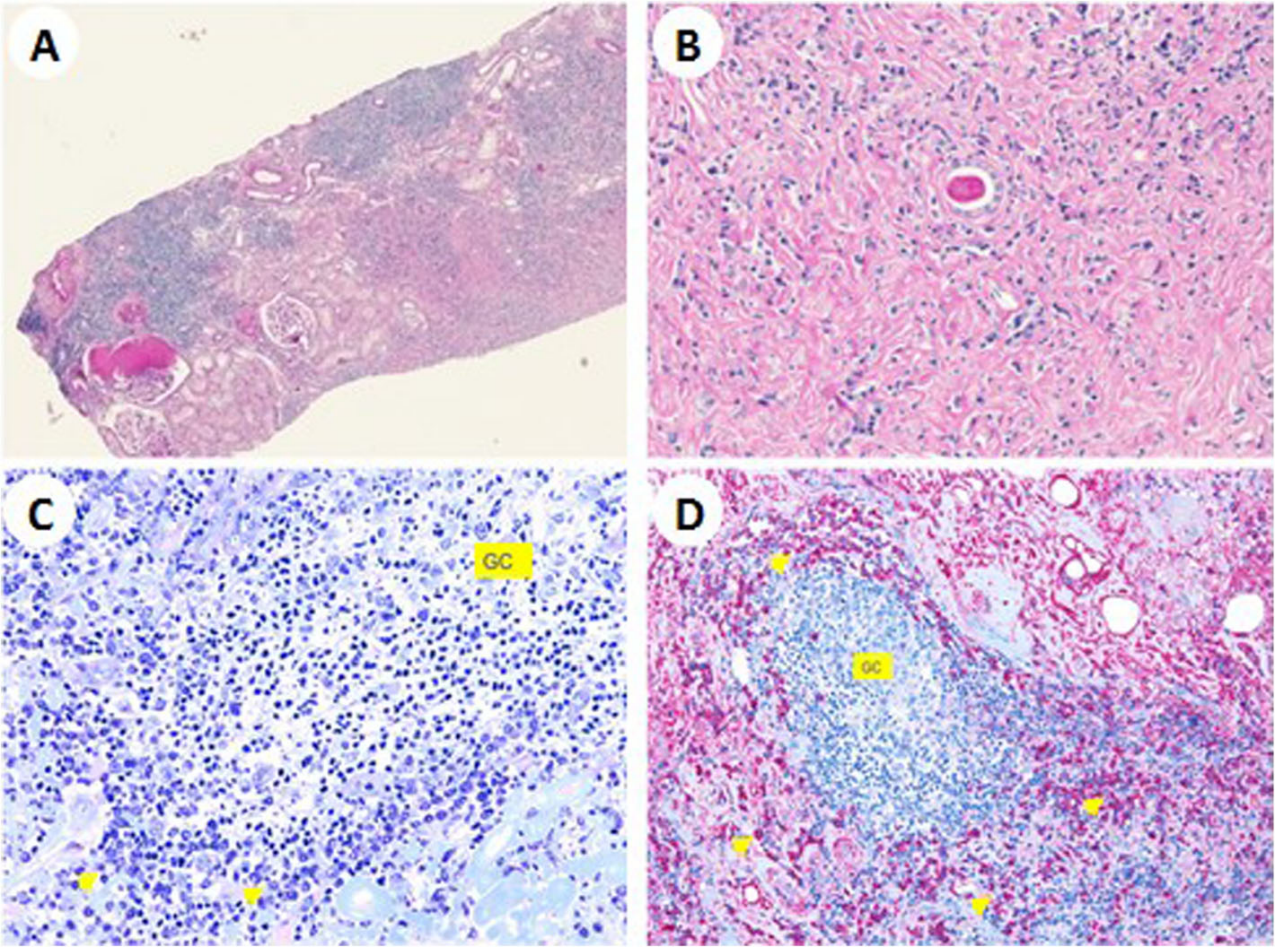
Diffuse tubulointerstitial fibrosis with two normal glomeruli (**a**); lymphoplasmatic infiltrate with parenchymal atrophy (**b**); a lymphoid follicle with a reactive germinal centre surrounded by mature plasma cells (yellow arrows) (**c**); fibrotic infiltration, Giemsa 40XIgG, IgG4-positive plasma cells (yellow arrows) (**d**)

The kidney biopsy revealed chronic nephritis with severe tubulointerstitial fibrosis, with no storiform pattern, and parenchymal atrophy along with lymphoplasma cells infiltrate (Fig. 2a, b); immunohistochemistry showed that 15–20% of IgG were IgG4 type. The Congo red test was negative. An inguinal lymph node biopsy highlighted follicular hyperplasia with plasmacytosis, with IgG4/IgG ratio > 50% (Fig. 2c, d).

**Fig. 3:**
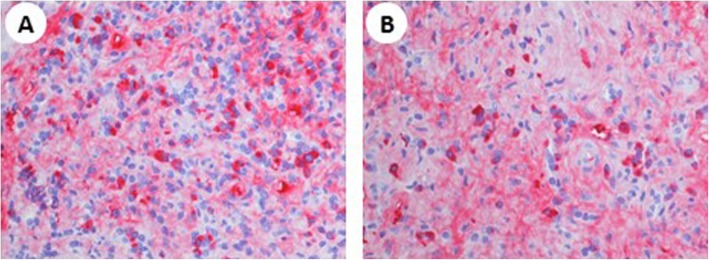
Dense lymphoplasmacytic infiltration with 14–16 infiltrating IgG4-positive plasma cells. **a** and **b** show two different high power fields at 400x magnification

We used the Japanese diagnostic criteria for IgG4-related disease published by Kawano et al. [8]. The patient was diagnosed with IgG4-RD in a definite way in view of the following:

- presence of some kidney damage: decreased kidney function, elevated serum IgG level, low serum complement levels, elevated serum IgE level (*criterion 1*);
- abnormal renal radiologic findings: multiple low-density lesions on enhanced CT (Fig. 1; *criterion 2*),
- elevated serum IgG4 level greater than 135 mg/dl (*criterion 3*);
- histologic findings in the kidney: dense lymphoplasmacytic infiltration with 14–16 infiltrating IgG4-positive plasma cells counted in different high power fields (HPF) at 400x magnification; two HPF are shown in Fig. 3a and b (*criterion 4a*);
- histologic findings in extra renal organs: follicular hyperplasia with plasmacytosis and IgG4/IgG ratio > 50% in lymph nodes (*criterion 5*), despite IgG4/IgG ratio of 15–20% in kidney specimen.

The patient was treated with methylprednisolone 0.6 mg/kg/day for 3 days, later tapering until oral methylprednisolone 16 mg daily. Since we saw a gradual improvement in laboratory data during the follow-up period, we prescribed oral methylprednisolone 4 mg daily for 6 months as maintenance steroid therapy. Plasma creatinine was 150.1 μmol/L, C3 level normalized and there was no albuminuria. We also noticed a net reduction of IgG4 from 1853 to 178.8 mg/dL.

**Fig. 4:**
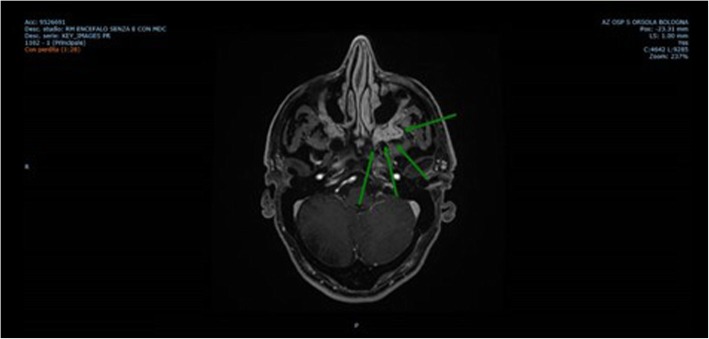
Pathological lesion biopsied (indicated by arrows) on Assial MRI 3DT1 fat saturated post-contrast sequence

**Fig. 5:**
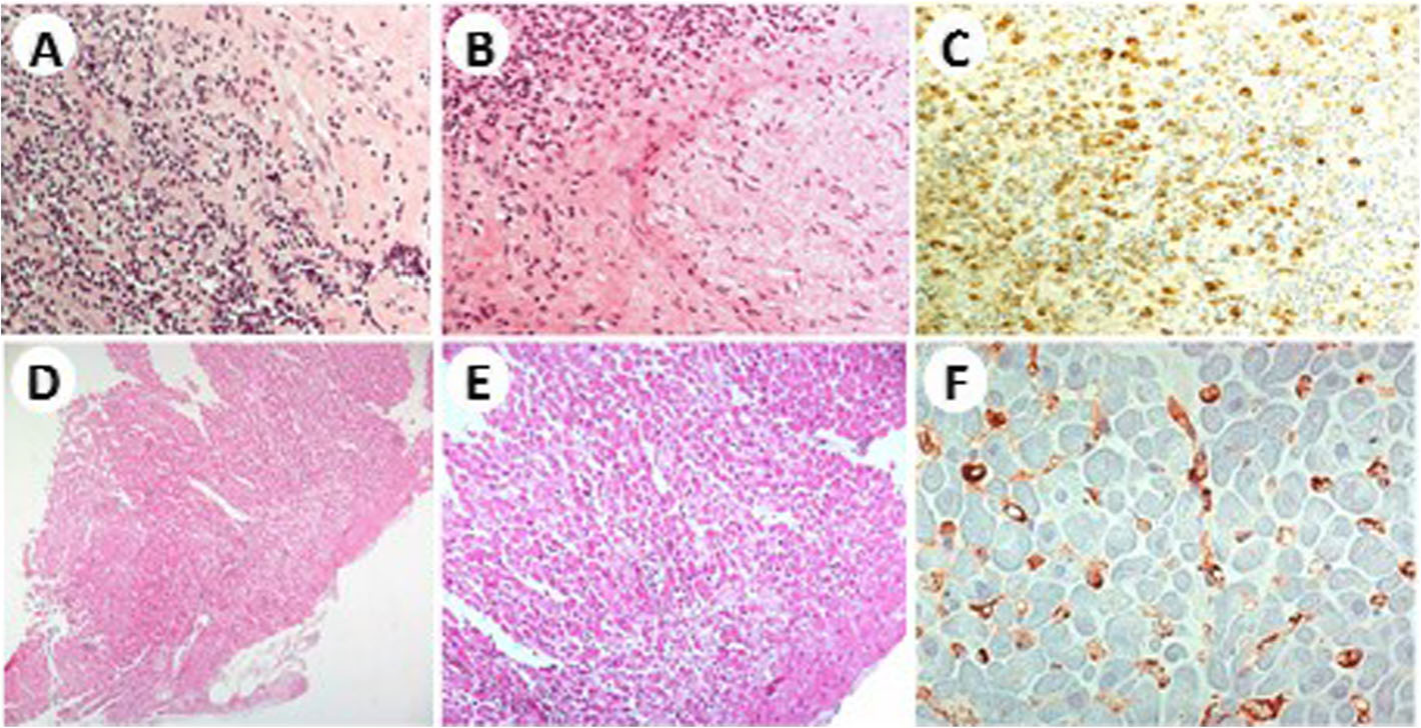
Intense fibrotic inflammation with a lot of plasma cells (**a**), involving a normal structure [a nerve on the left] (**b**) and IgG4+ plasma-cells staining (**c**). Diffuse and mild subendocardic and myocardic fibrosis (**d**), fibrosis with unspecific cellular alterations (**e**), perivascular IgG4+ plasma-cells deposition [< 30%] (**f**)

One year later, in spite of stable laboratory parameters and steroid maintenance therapy (4 mg/day), a new onset of tremors appeared associated to increased sialadenitis-related symptoms, left exophthalmos and left tinnitus. A cerebral MRI was performed which highlighted a pathological lesion with homogeneous enhancement on post-contrast studies, involving the left infratemporal fossa, the foramen rotundum and the inferior orbital fissure (Fig. 4). The patient was then submitted to a transsphenoidal endocranial biopsy of the pathological lesion that showed an intense fibrotic inflammation within the normal structure of a nerve with a plasma cell-rich infiltrate, mainly IgG4, as documented by immunohistochemistry (Fig. 5a, b, c).

Because of ECG alteration (right branch block and abnormal repolarization with pericarditis or possible ischemic signs) without cardiological symptoms, we performed an echocardiography. The patients showed normal left ventricular functions (EF 75%) with left and right ventricular wall thickness at the upper limits; no pericardial effusion could be detected. An endomyocardial biopsy, made to rule out any myocardial involvement, revealed the presence of unspecific subendocardial fibrosis without plasma cells (CD138 staining negative, not shown), as well as the presence of perivascular IgG4 positive plasma cells infiltrating cardiac tissue (Fig. 5d, e, f).

**Fig. 6:**
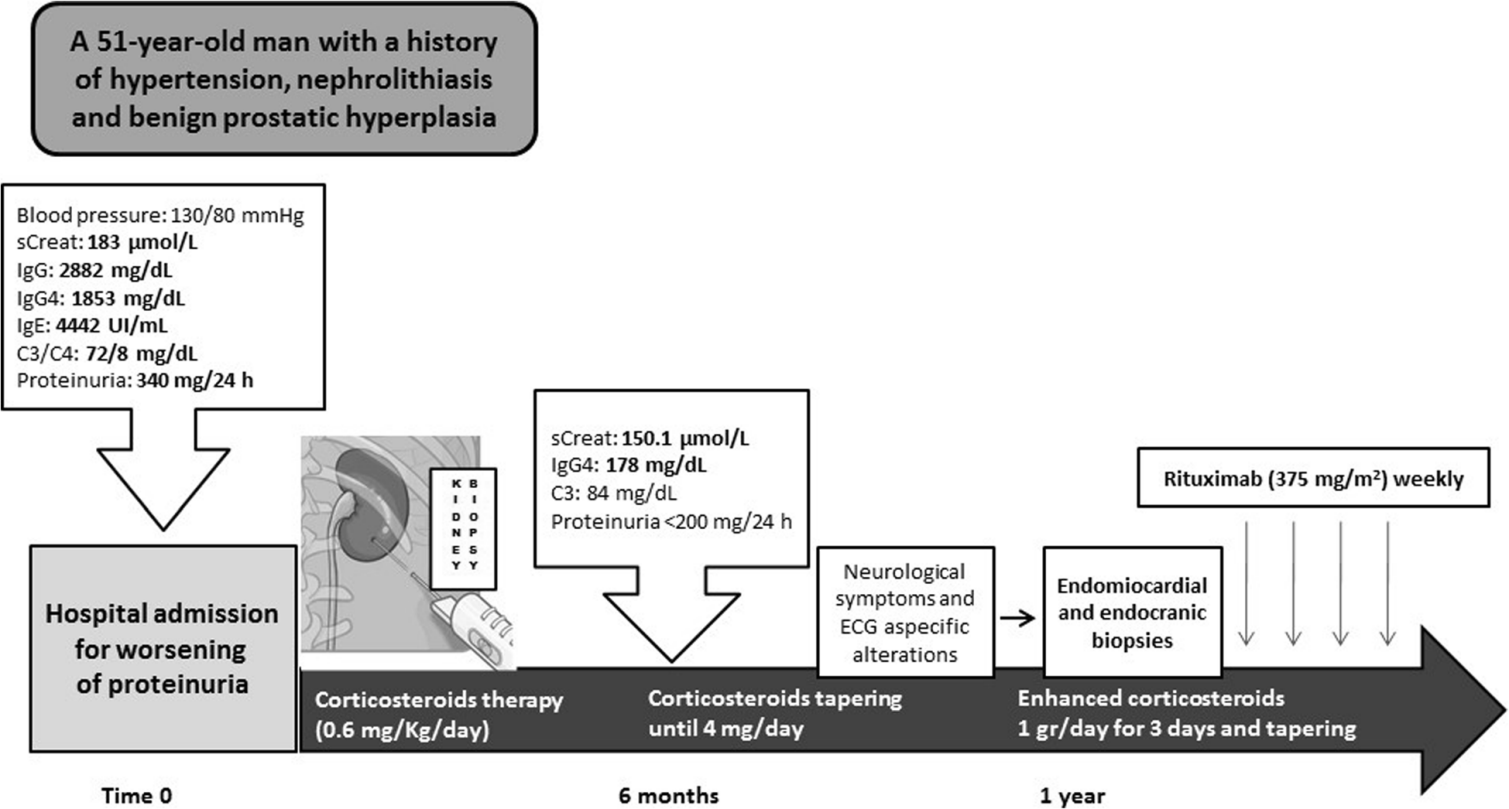
Timeline of the reported case. The laboratory values out of the normal ranges are highlighted in bold. sCreat, serum creatinine; ECG: Electrocardiography

We then prescribed a second-line therapy with steroid enhancement first, followed, after 1 month, by four Rituximab (RTX) weekly infusions (375 mg/m^2^ each one). The timeline of the case described here is detailed in Fig. 6.

After 1-year follow-up, the clinical conditions were stable; plasma creatinine was 152 μmol/L with a reduction of IgG and IgG4 (958 mg/dL and 93.3 mg/dL respectively).

## Discussion

The neurological manifestations of IgG4-RD could be related either to the direct injury of the central or peripheral nervous system or to the mass effect [6]. Various authors describe four different forms of neurological complications. IgG4-related orbital disease, in which IgG4-RD can implicate all extra-ocular muscles, levator palpebrae, structures emerging from the optic canal, or superior and inferior orbital fissure; according to the affected structure, different symptoms are described.

Goto et al. reported a case of IgG4-related ophthalmic disease misdiagnosed as intraocular tumor [10]. IgG4-RD could also occur as hypophysitis. The documentation of IgG4-RD-induced lesions of the pituitary gland introduces IgG4-related hypophysitis as a novel pathological entity, although plasmacytic/IgG4 involvement is already known in several other diseases, including both the more common lymphocytic-autoimmune and granulomatous pituitary disease and other rarer forms [11].

Moreover, IgG4-RD peripheral nerve disease has been also described, with symptoms caused both by the mass effect and by the contribution of the epineurium. Ohyama et al. reported the first case of IgG4-related neuropathy where the patient exhibited sensory and motor disturbances in the extremities; the authors showed the histopathological findings of a sural nerve biopsy, characterized by a clear thickening with abundant collagen fibers and infiltration of IgG4-positive plasma cells in the epineurium [12].

Finally, there are also cases of IgG4-RD meningeal disease. Among the most common causes of non-malignant meningeal inflammation, the disease can manifest in the substance of the dura mater, like pachymeninges of the brain or the spinal cord; rarely, IgG4-RD can affect the substance of pia mater (leptomeninges) without pachymeningeal involvement. In this condition, there is usually a mild lymphocytic pleocytosis in the cerebrospinal fluid. Parenchymal brain damages were found in a few patients, always associated with pachymeningitis [13].

The case described here appears to be peculiar, because it presents cerebral involvement without pachymeningitis, together with cardiac and renal parenchyma infiltrations.

There are three reports of IgG4-RD cases complicated by constrictive pericarditis [14–16]: in all of them, the diagnosis was histological through both autopsy and pericardiectomy. Mori et al. published a case of IgG4-RD in which autoimmune pancreatitis was associated with pericardial thickening diagnosed by contrast-enhanced CT scan of the chest [17]. In another case report, the pericardial involvement was identified through IgG4-positive plasma cells in pericardial effusion [18]; only a few studies have illustrated cytological examination in patients with IgG4-RD [19]. Vascular lesions, especially aortitis and periaortitis, represent further well-represented cardiological manifestations of IgG4-RD [20]. The affection of coronary arteries has also been described: Patel et al. have recorded a case of a 53-year-old Hispanic man who was sent to the emergency center and diagnosed with sudden cardiac death secondary to systemic IgG4-RD, with injury of coronary arteries [21].

IgG4-RD presents features appearing heterogeneously associated and it is not known how exactly the different organs are implicated [1, 6, 10, 11, 20–22].

IgG4 is the least abundant IgG subclass (< 5% of the total IgG); approximately 50% of IgG4 molecules consist of heavy chains that are weakly linked by non-covalent forces. Following the dissociation of these bonds, the chains recombine randomly creating asymmetric bispecific antibodies with two different antigen-binding sites [23]. Both B and T-cells are central in IgG4-RD pathogenesis, as demonstrated by the efficacy of B-cell depletion therapy [3, 4].

Firstly, activated IgG4+ B-cells and plasmablasts could enhance IgG4-RD both directly, with the production of IgG4-autoantibodies, and indirectly, through the activation of pathogenetic CD4 + T-cells. During the different stages of IgG4-RD, somatic hypermutation of IgG4+ plasmablasts suggest the continuative role of CD4+ T-cells in the pathogenesis of IgG4-RD [6]. At the beginning, dendritic cells or B-cells present antigens to T-lymphocytes in lymph nodes; at the same time, local signs from the innate immune system might determine the T helper cell polarization and their differentiation into effector or memory T-cells. Activated B-cells migrate to germinal centers and undergo somatic hypermutation, differentiating into memory B-cells or plasmablasts. The underlying mechanisms might be that, among CD4+ T-cells, both Th2 and regulatory T-cells, sustained by putative autoreactive B-cells, may drive collagen deposition and induce IgG4 class-switch and plasmablast expansion [23]. It is important to underline that, because of the absence of CD20 receptor on plasmablasts, RTX interferes with the principle B-cell/T-cell cross-talk process, which could explain the effectiveness of the therapy with RTX.

## Conclusions

This case report shows how the diagnosis of IgG4-RD is often challenging. In this particular instance, the patient presented a slow deterioration of renal function in concomitance with a series of other factors potentially causing chronic renal damage: high blood pressure, immunological and alloreactive profile.

Respiratory symptoms, such as hypertrophy of submandibular and paratracheal lymph nodes, allergic rhinitis and/or conjunctivitis, must be considered as an alarm bell. Unfortunately, the proteinuria is not a characterizing factor, and the renal signs appeared very faded. The neurological involvement might present itself in many different ways, and it can sometimes be distinguished by one significant parenchymal infiltration, like in this case, causing annoying and unremarkable symptoms, as tremor and tinnitus. The heart involvement shows atypical aspects and it can be asymptomatic in the initial phases. The early diagnosis, although difficult, is fundamental for a positive outcome.

Even if the patient initially responded to steroid therapy, he underwent disease relapse when doses were tapered, and our experience agrees with previous findings indicating that steroids often are not enough to control the disease [24].

RTX seems to be the most effective second-line treatment, although randomized controlled trials are still lacking.

## Data Availability

The datasets generated and/or analysed during the current study are not publicly available due [for data privacy and security reasons, the EHRs can be viewed upon authorized access through credentials by patients and providers], but are available from the corresponding author on reasonable request.

## Abbreviations

EF: Ejection fraction
IFN-γ: Interferon gamma
IgG4-RD: IgG4-Related Disease
IL-10: Interleukin 10
IL-1β: Interleukin 1 beta
IL-4: Interleukin 4
RTX: Rituximab
TGF-β: Transforming growth factor beta
